# Extracellular vesicles derived from host and gut microbiota as promising nanocarriers for targeted therapy in osteoporosis and osteoarthritis

**DOI:** 10.3389/fphar.2022.1051134

**Published:** 2023-01-06

**Authors:** Kenneth Chat Pan Cheung, Ma Jiao, Chen Xingxuan, Jia Wei

**Affiliations:** ^1^ Hong Kong Traditional Chinese Medicine Phenome Research Center, School of Chinese Medicine, Hong Kong Baptist University, Hong Kong, China; ^2^ Shanghai Jiao Tong University Affiliated Sixth People’s Hospital, Shanghai, China

**Keywords:** osteoporosis, gut microbiota, microbiota extracellular vesicles, microbiota-host communications, osteoarthritis, exosome

## Abstract

Osteoporosis (OP), a systemic bone disease that causes structural bone loss and bone mass loss, is often associated with fragility fractures. Extracellular vesicles (EVs) generated by mammalian and gut bacteria have recently been identified as important mediators in the intercellular signaling pathway that may play a crucial role in microbiota-host communication. EVs are tiny membrane-bound vesicles, which range in size from 20 to 400 nm. They carry a variety of biologically active substances across intra- and intercellular space. These EVs have developed as a promising research area for the treatment of OP because of their nanosized architecture, enhanced biocompatibility, reduced toxicity, drug loading capacity, ease of customization, and industrialization. This review describes the latest development of EVs derived from mammals and bacteria, including their internalization, isolation, biogenesis, classifications, topologies, and compositions. Additionally, breakthroughs in chemical sciences and the distinctive biological features of bacterial extracellular vesicles (BEVs) allow for the customization of modified BEVs for the therapy of OP. In conclusion, we give a thorough and in-depth summary of the main difficulties and potential future of EVs in the treatment of OP, as well as highlight innovative uses and choices for the treatment of osteoarthritis (OA).

## 1 Introduction

Osteoporosis (OP), a systemic bone disease that causes increased bone fragility and fracture susceptibility, is characterized by a decrease in bone mass and the loss of bone microstructure (Prestwood et al., 1995; Brown, 2017; Liu et al., 2022a). The two most prevalent kinds of primary OP are postmenopausal OP, caused by an inadequate estrogen supply and senile OP brought on by aging (Ensrud and Crandall, 2017). Around 200 million individuals worldwide have OP, and the most prevalent problem, OP fracture, affects 20% of men and 50% of postmenopausal women during their lifespan. This significantly reduces patients’ lifestyle quality and puts a heavy economic strain on society (Black and Rosen, 2016; Chen et al., 2022a). In recent decades, numerous efforts have been made in the treatment of OP owing to its widespread occurrence and complications (Li et al., 2020). The dynamic equilibrium between bone synthesis (osteoblasts) and bone resorption (osteoclasts) has an impact on bone mass the most (Cui et al., 2020). Consequently, anabolic medications (such as synthetic parathyroid hormone) and antiresorptive therapies (such as calcitonin, bisphosphonates, denosumab, and estrogens) are being used to treat OP (Muraca and Cappariello, 2020; Hu et al., 2021). However, the potential applications of these drugs have been constrained by their bioavailability and long-term toxicity. Thus, it is an urgent requirement to generate and develop a more reliable and efficient approach to overcome OP.

## 2 Extracellular Vesicles (EVs): An innovative strategy to reverse osteoporosis

Pharmacological treatments for OP target to sustain homeostasis of the bone remodeling unit. Bone anti-resorptive and bone anabolic agents are available in clinical management.

### 2.1 Properties of EVs

EVs, a heterogeneous family of membrane-limited vesicles originating from the endosome or plasma membrane, can be divided into three types according to size (Abels and Breakefield, 2016). Apoptotic bodies, ranging from 800 to 5,000 nm, are produced by shedding cells during apoptosis, while microvesicles with a 200–2000 diameter are produced by the plasma membrane in a budding manner. Besides, exosomes (40–200 nm) are secreted from intracellular multivesicular bodies fused with the cytoplasmic membrane (He et al., 2021; Zhang et al., 2022). Among these EVs, exosome is the most critical one because of the therapeutic potential of exosomes from certain cell types (Lu et al., 2021a).

### 2.2 Epigenetic regulation and EVs

Epigenetics is the regulation of gene expression or phenotype without altering the sequence of the structural DNA and involves histone modifications, DNA methylation, and RNA-based mechanisms (Tzouvelekis and Kaminski, 2015; Yang et al., 2015; Yang et al., 2020a). Non-coding (nc) RNAs, including microRNAs (miRNAs), long non-coding (lnc) RNAs, and circular (circ) RNAs, are reported to play a significant role in epigenetic modifications (Muraca and Cappariello, 2020; Li et al., 2021). And ncRNAs can affect bone metabolism by regulating cell processes, including proliferation, differentiation, apoptosis, and autophagy. A study revealed that 260 circRNAs, 70 lncRNAs, and 13 miRNAs were differentially expressed between patients with postmenopausal OP and healthy controls (Jin et al., 2018). In addition, recent studies declared that ncRNAs could play an essential role in OP after delivering to recipient cells using EVs as a carrier (Cao et al., 2019; Tu et al., 2019; Zhang et al., 2022).

### 2.3 Different cell-derived EVs and osteoporosis

#### 2.3.1 MSC-derived EVs

Recently, mesenchymal stem cells (MSCs) have been regarded as potential approaches to treat different diseases based on their capacity for self-renewal, paracrine production, and immunomodulation. MSCs can be isolated from bone marrow, peripheral blood, adipose tissue, umbilical cord, dental pulp, endothelial polyps, placenta, and Wharton’s jelly (Seshareddy et al., 2008; Can and Balci, 2011; Abbaszadeh et al., 2020a; Abbaszadeh et al., 2020b; Huldani et al., 2022). Ge et al. revealed the osteogenic induction and osteoporotic effect of human umbilical cord MSC (HucMSC)-derived exosomes and found two critical miRNA (hsa-miR-2110 and hsa-miR-328-3p) which associated with bone differentiation (Yahao and Xinjia, 2021). According to Qi’s study, exosomes secreted by MSCs derived from human induced pluripotent stem cells (hiPSC-MSC-Exos) could increase mRNA and protein expression of (Osteoblast) OB-related genes in bone marrow MSCs derived from ovariectomized rats. Moreover, hiPSC-MSC-Exos significantly enhanced bone regeneration and angiogenesis in ovariectomized rats (Qi et al., 2016). Based on RNA sequencing results, Wharton’s jelly-MSC-EVs enriched miR-21, miR-29, miR-221, and let-7a, which were corrected to BMP and PI3K/AKT signaling pathways for osteoporotic treatment (Lu et al., 2021b). In addition, MSCs could regulate immune cells’ function, inflammation, and the microenvironment (Malekpour et al., 2022).

In patients with OP, sarcopenia is often associated with (Dai et al., 2022; da Silva et al., 2017; Yoshimura et al., 2017). Bone marrow mesenchymal stem cells (BMSCs), as multipotent cells with self-renewing activities, can differentiate along have osteogenic lineage in response to stimulation by environmental factors. In addition, the senescence and decreased osteogenic abilities of BMSCs play critical roles in OP (Zhang et al., 2020a; Dai et al., 2022). EVs are regarded as an intrinsic systematic delivery vehicle by transporting cargos of ncRNAs, lipids, and proteins, especially miRNAs which are important for various biological processes such as bone formation, resorption, remodeling, and bone cell differentiation (Zhang et al., 2020a; Zhang et al., 2020b; Dai et al., 2022).

According to previous studies, miR-22-3p delivered by BMSC-derived EVs could induce inhibition of the MYC/PI3K/AKT pathway to promote osteogenic differentiation (Zhang et al., 2020a). Peng et al. also found that BMSC-EVs could deliver miR-196a to enhance osteoblastic differentiation by activating the Wnt/β-catenin pathway (Peng et al., 2021). In addition, miR-935, miR-29a, miR-34a, miR-34c, miR-31a-5p, and miR150-3p could also alleviate OP progression (Xu et al., 2018; Sadanand Fulzele et al., 2019; Yang et al., 2019; Lu et al., 2020; Zhang et al., 2021a; Qiu et al., 2021).

#### 2.3.2 OC- and OB-derived EVs

OP, as a metabolic disorder, is induced by the imbalance of bone remodeling (Cao, 2011; Deng et al., 2015; Yuan et al., 2018). Bone remodeling is accomplished *via* the precise coordination of the activities of the two specific cells: bone-forming osteoblasts and bone-resorbing osteoclasts (Yuan et al., 2018). The crosstalk between (osteoclasts) OCs and (osteoblasts) OBs is critical in the regulation of bone homeostasis (Yuan et al., 2018). Previous studies revealed that OC could regulate OB activity either by direct cell-cell contact or the secretion of cytokines. Thus, in recent years, many researchers have also put focused on OB and OC derived EVs (Holliday et al., 2017; Chen et al., 2018; Yuan et al., 2018; Zhu et al., 2020a).

A study demonstrated that EVs isolated from OCs are paracrine regulators of osteoclastogenesis. OCs express RANKL on their surface. And RANKL binds with RANK on the surface of monocytes to stimulate the differentiation of monotype into OCs. Whereas OBs express OPG, which binds RANKL to inhibit its binding to RANK (Huynh et al., 2016). Deng et al. showed that RANKL enriched-EVs are generated from OCs and stimulated OC formation by engagement of RNAK (Deng et al., 2015). Yang et al. showed that OC-derived miR-23a-5p-containing exosomes could efficiently suppress osteogenic differentiation by inhibiting Runx2 and promoting YAP1-mediated MT1DP (Yang et al., 2020b). Sun et al. reported that OCs secrete microRNA-enriched exosomes, by which miR-214 is transferred into OBs to inhibit their function. In addition, OC-specific miR-214 transgenic mice expressed higher circulating miR-214 and downregulated OC activity (Sun et al., 2016). Moreover, another study also confirmed that OC-derived exosomal miR-214-3p could mediate osteoclast-to-osteoblast communication by inhibiting osteoblastic bone formation (Li et al., 2016a).

For EVs from OBs, Wei et al. demonstrated that EVs released by OBs at the mid-to-late differentiation stage markedly enhanced osteogenesis (Wei et al., 2019). Cui et al. demonstrated that mineralizing OB-derived exosomes could obviously influence miRNA profile and partially cause a change in the expression of miRNA in recipient ST2 cells (Wang et al., 2021). Importantly, Luo et al. found that senescent OB-derived exosome-mediated miR-139-5p regulated endothelial cell function (including upregulation of senescence and apoptosis and inhibition of proliferation and migration) *via* the exosomal pathway (Lu et al., 2021c).

#### 2.3.3 Osteocyte-derived EVs

Except for BMSCs, OCs, and OBs, bone-derived EVs can also be secreted by osteocytes (Lyu et al., 2020). Osteocytes, as mechanosensitive cells, have regulatory effects on loading-induced bone formation *via* the secretion of paracrine factors (Eichholz et al., 2020). Another research showed that exosomes secreted by MLO-Y4 osteocyte cells exposed to mechanical strain (Exosome-MS) contributed to HPDLSC proliferation and osteogenic differentiation through PTEN/AKT and BMP2/Runx2 pathways. Moreover, these exosomes expressed higher miR181b-5p which is closely related to cell proliferation, apoptosis, and immune inflammation (Liu et al., 2019; Wang et al., 2019; Lv et al., 2020). Similarly, Eichholz et al. illustrated that osteocyte-derived EVs with pro-osteogenic potential could enhance bone regeneration and repair in diseases such as OP (Eichholz et al., 2020).

#### 2.3.4 Macrophage-derived EVs

Macrophages (Mφs), as an important part of innate immunity, depending on the environment, can be divided into two types: the anti-inflammatory phenotype (M2) and the pro-inflammatory phenotype (M1). As we know, chronic inflammation is one of the direct inducers of OP (Ginaldi et al., 2005; Kong et al., 2019; Liu et al., 2021a; Yu et al., 2021). According to one study, osteolysis occurs in mice when Mφs become polarised toward the M1 phenotype (Gao et al., 2021). Based on microarray analysis, miR-98 is a candidate cargo of the M1 Mφs-EXOs and led to bone loss and OP progression through the DUSP1/JNK pathway (Yu et al., 2021). There is research revealed that all Mφ subtypes (M0, M1, and M2) can promote the osteogenic differentiation of BMSCs, and M1 Mφs may be critical to the early phases of osteo-induction and bone regeneration, while M2 Mφs may foster continued bone regeneration (Chen et al., 2020a; Kang et al., 2020; Liu et al., 2021a; Song et al., 2022a).

Exosomes can be released by Mφs cells during the regulation of inflammatory responses. Liu et al. demonstrated that both M1 and M2 Mφ-exosomes upregulated osteogenesis of BMSCs (Liu et al., 2021a). One miRNA sequencing analysis of the exosomes showed that Mφs-derived exosomal miR-3473e plays a pivotal role in the promotion of osteo-/angio-genesis *via* upregulation of the Akt1 gene (Wang et al., 2022a). Interestingly, Zhang et al. found the miR-144-5p levels were highly enriched in exosomes derived from bone marrow-derived macrophages in type 2 diabetes, and it could be transferred into BMSCs to regulate bone regeneration *via* Smad1 (Zhang et al., 2021b).

M1 Mφ-exosomes promote osteogenesis of BMSCs through microRNA-21a-5p at the early stage of inflammation (Liu et al., 2021a). Moreover, miR-5106, miR-26a-5p, and miR-22-3p derived from M2 Mφ-exosomes are also reported as targets to promote osteogenic differentiation in BMSCs and MSCs (Xiong et al., 2020; Bin-Bin et al., 2022; Liu et al., 2022b). Besides, Mg^2+^-mediated Mφs could promote the osteogenic differentiation of BMSCs *via* the autophagy pathway by reducing miR-381 in macrophage-derived exosomes (Zhu et al., 2022).

#### 2.3.5 Endothelial cell-derived EVs

Research show abnormal angiogenesis and excessive osteoclastic activity would encourage aberrant bone resorption that is connected to OP (Kusumbe et al., 2014; Ramasamy et al., 2014; Fan et al., 2017; Farr et al., 2017; Song et al., 2019). It was reported that there is a positive correlation between blood vessel density and osteogenesis adversely associated with bone resorption (Sivaraj and Adams, 2016; Huang et al., 2018). Endothelial cells (ECs) are located in the inner layer of the vascular vessel and are frequently secreting substances due to their active properties (Seton-Rogers, 2014). Studies have further shown that EC-exos contain a variety of chemicals relevant to cell migration, proliferation, and vascular formation. EC-exos could suppress OP progression *via* delivering miR-155 (Todorova et al., 2017; Song et al., 2019). Wilner et al. showed that EVs containing miR-31 were secreted from senescent ECs and presented an inhibitory effect on osteogenic differentiation by counteracting the Frizzled-3 gene (Weilner et al., 2016). In addition, EC-Exos delayed the progression of glucocorticoid-induced OP by inhibiting apoptosis and ferroptosis in dexamethasone-stimulated OBs (Yang et al., 2021).

As reported, endothelial progenitor cells (EPCs) can also influence osteoclastogenesis *via* EVs. Evs derived from EPCs could prevent glucocorticoid-induced OP in mice by suppressing the ferroptosis pathway in OBs (Cui et al., 2019; Lu et al., 2019). Besides, Cui et al. illustrated that EPC-exos promoted osteoclastogenesis by the Lnc-MALAT-1/miR-124 pathway (Cui et al., 2019).

## 3 Gut microbiota

In recent years, the study of microbial EVs has also begun to receive attention. The skin and mucosal surfaces of the human body are colonized by a large number of microorganisms, which is particularly important in the gut. Our understanding of the connection between gut microbiota (GM) and human health has considerably increased because of the National Institutes of Health-funded Human Microbiome Project (Lloyd-Price et al., 2017). Along the length of the gastrointestinal tract, a complex, dynamic microbial community, including bacteria, viruses, archaea, and eukaryotes, reaches its highest density in the colon (Lozupone et al., 2012). In the human stomach, 1,150 different bacterial species have been found, with 160 different species on average for each person (Lozupone et al., 2012). More than 70% of the gut bacteria belong to the two phyla *Bacteroidetes* and *Firmicutes*, other phyla such as *Proteobacteria, Verrucomicrobia, Fusobacteria*, and *Actinobacteria* are only present in small amounts (Eckburg et al., 2005; Sultan et al., 2021).

The gut flora has a substantial impact on human health. They play a crucial part in the growth of the immune system (Lee and Mazmanian, 2010). Additionally, they break down indigestible plant fibers to produce vital metabolites like short-chain fatty acids (SCFAs) (Tremaroli and Backhed, 2012). Clostridial clusters IV and X1Va, *Bacteroides*, and Bifidobacterium, dominate the SCFA production (Martens et al., 2011; Sultan et al., 2021). The three main SCFAs produced by the microbiota are propionate, acetate, and butyrate. In colonocytes, butyrate serves as the predominant energy source, while acetate and propionate are substrates for lipogenesis and gluconeogenesis in peripheral tissues (Wolever et al., 1991; Mottawea et al., 2016). SCFAs also regulate how well the gut barrier is functioning. For instance, butyrate increases the production of proteins linked to tight junctions (Bordin et al., 2004). In addition to the colonic fermentation of dietary fibers, the control of the metabolism of choline, bile acid metabolism, and insulin resistance are a few other host metabolic pathways that the GM interacts with (Tremaroli and Backhed, 2012).

**FIGURE 1:**
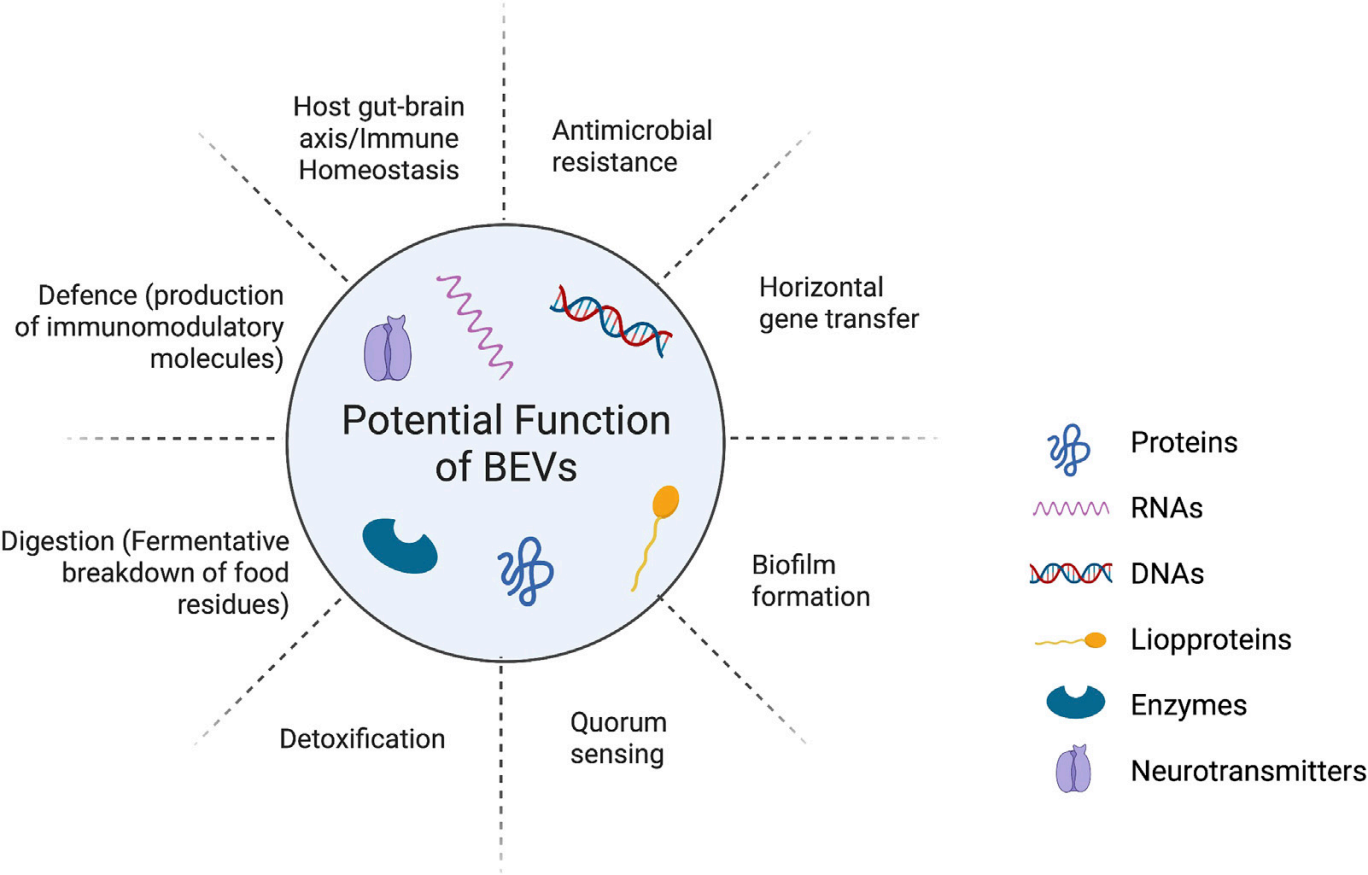
The roles of bacterial extracellular vesicles (BEVs) on the health of the host as a vehicle for moving bioactive payload (such as miRNA, DNA, mRNA, proteins, lipids, and carbohydrates) and their potential function.

The host senses bacterial metabolites as part of the microbiota-host interaction. Importantly, studies demonstrate that human commensal EV mediates immunological control and disease prevention (Shen et al., 2012). We highlight what is currently known about the roles of bacterial extracellular vesicles (BEVs) on the health of the host as a vehicle for moving bioactive payload (such as miRNA, DNA, mRNA, proteins, lipids, and carbohydrates), as well as their potential function as molecular pathways in Figure 1.

## 4 Bacterial EVs (BEVs)

In the intestinal ecosystem, the two-way communication between microbiota and host does not involve direct cellular contact. Both microbiota and host-derived EVs are key players in this interkingdom crosstalk. There is now growing evidence that bacterially secreted vesicles mediate microbiota function by translocating and delivering effector molecules that regulate host signaling pathways and cellular processes into host cells. Emerging evidence suggests that GM is essential for maintaining bone homeostasis and preventing the onset of OP (Xu et al., 2017). According to their contribution to preserving human health, gut microorganisms can be classified as harmful bacteria (probiotics), beneficial bacteria, and neutral bacteria (Gentile and Weir, 2018). Probiotics are recognized as live microorganisms by the Food and Agriculture Organization of the United Nations (FAO) and the World Health Organization (WHO). They are beneficial for the host when supplied in sufficient numbers. For example, probiotics have been demonstrated to reduce OP (Song et al., 2022b). Notably, direct cell interactions are not necessary for probiotics and hosts to communicate. There is mounting evidence that these probiotic EVs modify host signaling pathways and deliver bioactive materials to host cells to control the function of distant organs (Liu et al., 2022a; Chen et al., 2022b; Liu et al., 2022c; Liu et al., 2022d).

BEVs are non-reproducible phospholipid bilayer nanocarriers that are released by most bacteria. They provide communication between bacteria and their hosts by providing various compounds involving metabolites, proteins, lipids, and nucleic acids. As a result, they have a significant impact on the regulation of physiological and pathological processes (Zihao Ou et al., 2022). Owing to their nanosized dimensions, drug loading capacity, cell-free systems, low toxicity, and superior biocompatibility, BEVs, particularly probiotic EVs, have developed into an effective platform for biological applications (Witwer et al., 2021). The advantages of rapid bacterial growth and maturation in high-density cultures (fed-batch fermentation) allow for large-scale manufacture of BEVs, compared to the low extraction rates of mammalian EVs (MEVs), which are very low (Liu et al., 2018; Liu et al., 2020a; Liu et al., 2020b). Additionally, breakthroughs in synthetic biology and the distinctive biological pathways of BEVs allow for the customization of modified BEVs for the therapy of OP (Service, 2014; Toyofuku et al., 2019). Thus, the gut-bone axis system may be significantly impacted by the use of both natural and engineered BEVs, which would effectively manage the onset and progression of OP. At the same time, there are some difficulties with the clinical application of EVs, mainly in terms of yield and targeting. It is difficult to obtain pure exosomes from natural sources, and there is a wide range of exosome sources. Whether all exosomes from various sources can be obtained by a continuous extrusion of cells to obtain exosome-mimicking nanovesicles and whether their structural integrity and physiological activities are the same as those of natural exosomes still need to be further investigated. Although progress has been made in targeting modifications, the *in vivo* environment is complex, and it is uncertain whether the modified exosomes will still have the desired targeting properties once they enter the body. Therefore, targeting modifications of exosomes is still a major focus of research.

BEVs are lipid nanostructures (about 25–300 nm) derived from parental bacterial cells and are found not only in prokaryotes but also in all living domains, such as fungi, protozoa, and plant cells. We illustrate the biogenesis and composition of EVs derived from eukaryotic host cells (host EVs), gram-negative bacteria (outer membrane vesicles, OMVs), and gram-positive bacteria (membrane vesicles). Generation of EVs by host cells can occur *via* outward budding of the plasma membrane, resulting in microvesicles. Alternatively, inward budding of the endosomal membrane results in multivesicular endosomes (MVEs) with intraluminal vesicles (Jung et al., 2021). They contain a variety of molecular components such as nucleic acids (DNA, RNA), proteins, lipids, and other organic substances. More recent studies have shown that the production of BEVs can be achieved by different biogenetic mechanisms, with some EVs possibly originating from blistering cells and others being secreted during cell lysis. Each of these groups of EVs contains a “cargo” from the cell’s source: a membrane-rich cargo in the blistering form and a membrane- or cytoplasm-rich cargo in the lysed form. In this way, the primary mechanism for the biogenesis of BEVs is regulated by the cellular response to the environment and therefore affects the composition of BEVs. Table 1 summarizes the overlapping and distinguishing characteristics of these EV populations.

**TABLE 1:** Overlapping and distinguishing characteristics of EV populations (Jung et al., 2021).

Features	Host EVs	OMVs	Membrane vesicles
Cellular source	All eukaryotic cell types	Gram-negative bacteria	Gram-positive bacteria
Subcellular source	Plasma membrane or multivesicular endosomes	Outer membrane	Plasma membrane
Size (diameter)	30–1,000 nm	20–250 nm	20–250 nm
Structure	Spherical nanostructures enclosed by membrane		
Release	Homeostasis and during infections		
Composition	Adjusts upon environmental terms		
Role	Intra- and inter-organism/species interactions		
Route of isolation	For example, size exclusion chromatography or ultracentrifugation		

## 5 BEVs and immune homeostasis

The gut microbiota is considered a “hidden organ” because the products encoded by the microbiota actively contribute to many essential host functions. In addition to its role in nutrition, metabolism and energy production, the gut microbiota also regulates immune homeostasis. EVs (especially BEVs) generated from GM are crucial for preserving gut immunological homeostasis. A cascade of immune signaling is triggered by the interaction of BEVs with pattern recognition receptors like NOD1, NOD2, and TLR on immune cells. BEVs contain various copies of microorganism-related molecular patterns, such as LPS, RNA, DNA, periplasmic proteins, and peptidoglycan (Kaparakis et al., 2010; Soult et al., 2013; Bielaszewska et al., 2018; Meganathan et al., 2020). Notably, the interaction between immune cells and EVs cargo is related to virulence and the parent stain. Proteomic research has shown that the TLR 2 lipoprotein agonist is only present in EVs from virulent *mycobacterium* strains (Prados-Rosales et al., 2011). Additionally, the TLR-EV interaction is receptor-specific. For instance, the cellular responses of TLR 2/1 and TLR 4 were upregulated, but those of TLR 2/6 were suppressed, while TLR 5 was unaffected by the EVs generated by the *Lactobacillus* and *Bifidobacterium genera* (van Bergenhenegouwen et al., 2014). Besides, EVs’ sRNA and miRNA content have the potential to suppress the immune system; in the instance of sRNA from the fungus Botrytis cinerea, which silences genes to reduce plant immunity (Weiberg et al., 2013). Additionally, anopheline mosquito-produced microRNA (miRNA) may interfere with host miRNA and modulate some immune responses (Arca et al., 2019), suggesting that EVs would be used as a strategy by pathogens to suppress the host immune system (Lee, 2019).

## 6 The use of natural BEVs in OP treatment

Over the past 10 years, the interaction between GM and OP has drawn a lot of attention (Zhang et al., 2021c; Wang et al., 2022b). The GM, in particular probiotics, has been identified as a potential target for the treatment of OP, such as *Lactobacillus rhamnosus GG*, *Lactobacillus reuteri, Lactobacillus paracasei, Bifidobacterium longum, and Akkermansia muciniphila* (AKK) (Thomas et al., 2012; Ohlsson et al., 2014; Parvaneh et al., 2015; Li et al., 2016b; Liu et al., 2021b). Given the importance of GM in maintaining bone homeostasis, examining the ways in which it interacts with bone can serve as a therapeutic platform for translational medicine. Recently, it has been demonstrated that the secretion of BEVs, which may safely carry numerous bioactive chemicals to distant tissues/cells, is essential for the communication of signals between bacterial and mammalian cells (Chen et al., 2019; Chen et al., 2020b; Chen et al., 2022b). As a result, researchers have focused more on determining if BEV made from GM is an important mechanism for the GM-induced regulation of OP.

Excitingly, a novel treatment approach called Fecal Microbiota Transplantation (FMT) delays the progression of degenerative and chronic disorders (Hanssen et al., 2021). According to Xie et al., replacing aged GM (EGM) with young GM (CGM) prevented the loss of bone strength and bone mass in ovariectomy (OVX)-induced OP mice (Liu et al., 2021b). Transplanting CGM dramatically enhanced the amount of AKK in OP mice, according to 16S rRNA sequencing. Additionally, AKK given directly to OVX-induced mice dramatically increased OP. Further investigation revealed that administration of GW4869, a neutral sphingomyelinase inhibitor, interferes with the secretion of BEVs, and the anti-osteoporotic effects of AKK were dramatically reduced.

**FIGURE 2:**
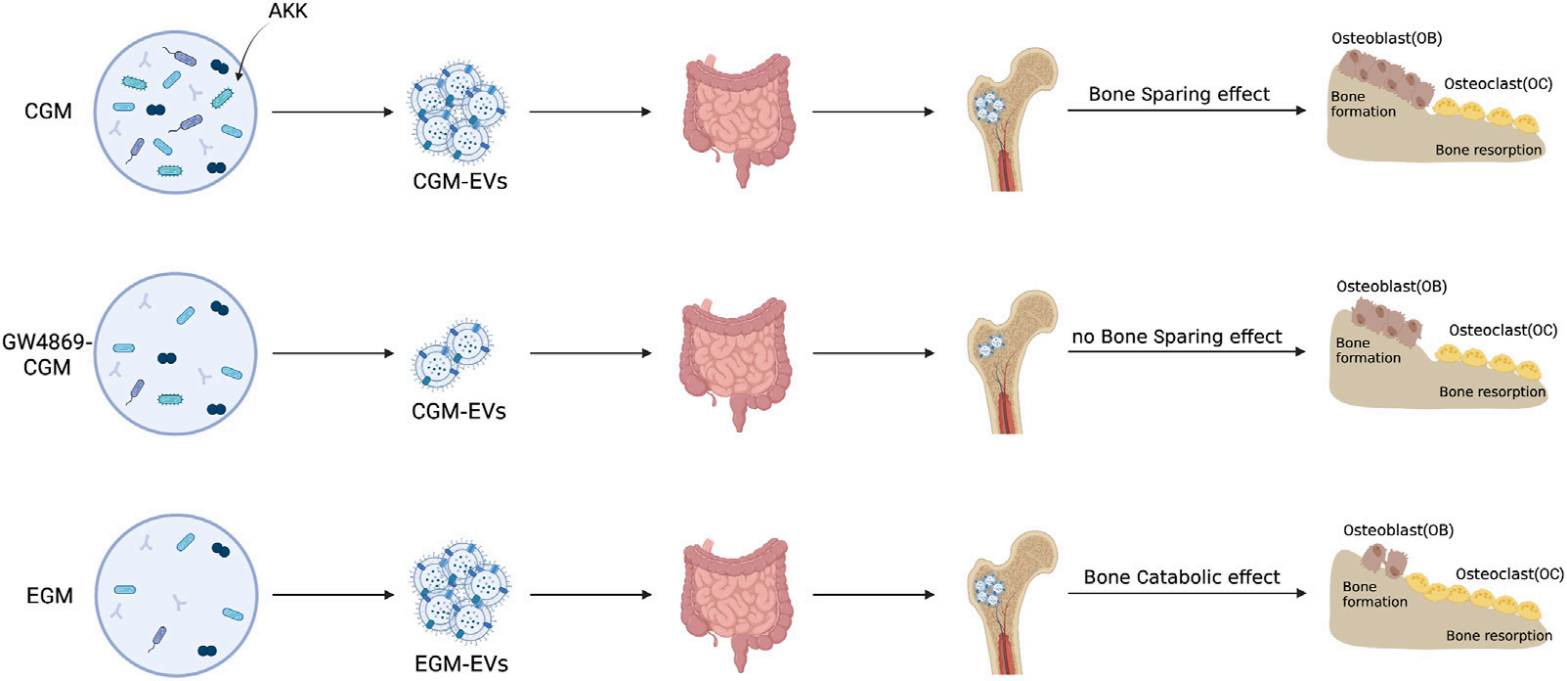
OP is treated with natural BEVs. The direct intervening OVX animals shown in the schematic diagram may operate similarly to their parent bacteria in promoting osteoblast growth and suppressing osteoclast activity, suppressing the loss of bone mass and strength, and the degeneration of the bone microarchitecture. Inhibitors of neutral sphingomyelinases, such as GW4896, are used to treat the gut microbiota of youngsters and the elderly (Liu et al., 2021b).

Accordingly, the intervention of OVX mice with BEVs derived from AKK can have effects on OBs that are similar to those of their parent bacteria, promoting OB growth and inhibiting OC activity, suppressing the loss of bone mass, strength and the deterioration of the bone microarchitecture **(** Figure 2 **)**. Although Xie et al. did not examine the precise essential mechanisms by which BEVs produced from AKK enhance OP, multi-omics sequencing of BEVs could help to solve this problem and modifying the development of future modified BEVs.

**FIGURE 3:**
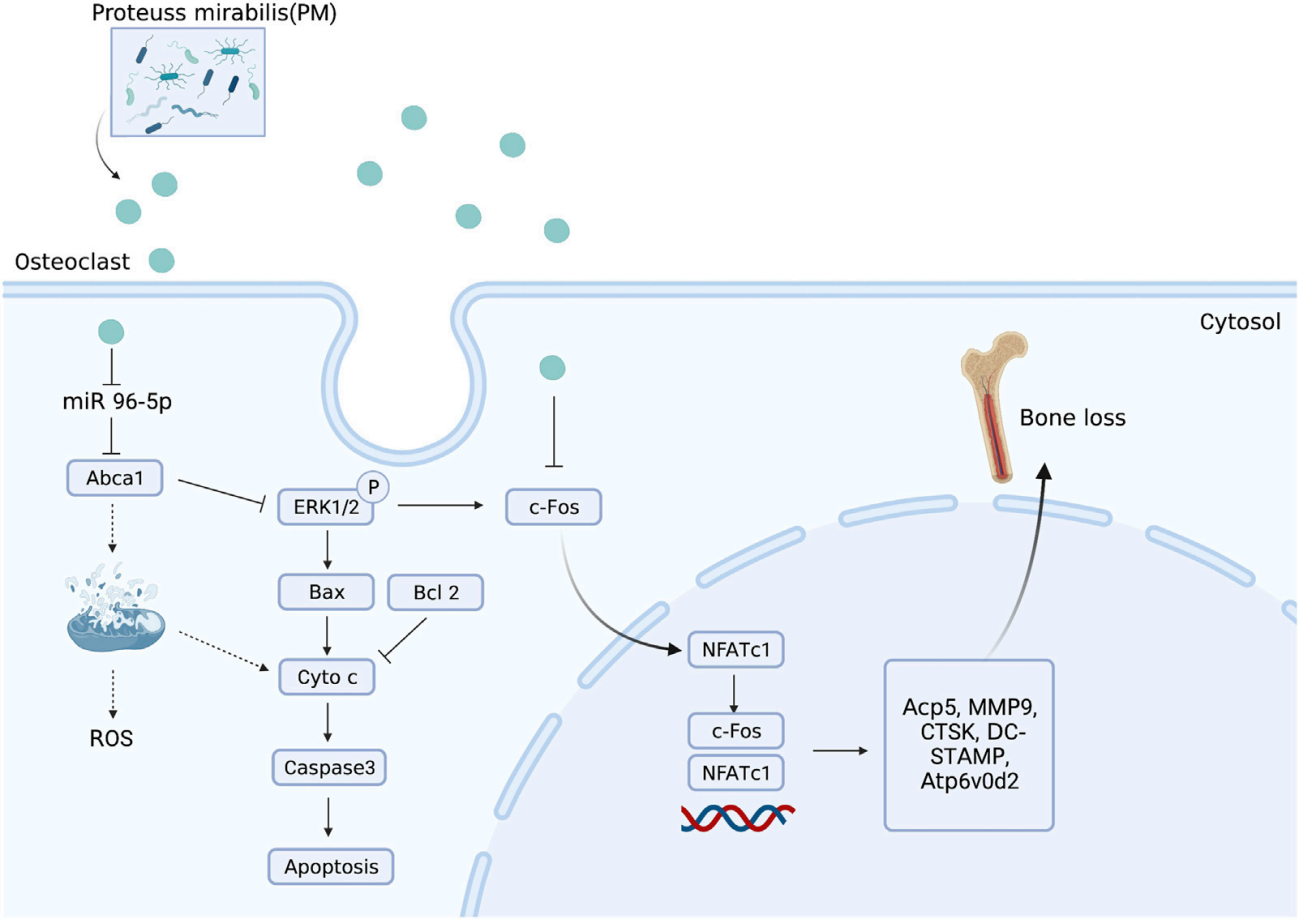
BEVs produced from *Proteus mirabilis* alleviate bone loss in OP. Administration of PM-derived BEVs reduced bone loss in OVX-induced OP mice *via* the regulation of miR-96-5p/Abca1 pathways.

Nandakumar et al. recently showed that BEVs produced from the intestinal strain *Proteus mirabilis* (PM) suppress osteoclastogenesis and bone resorption (Wang et al., 2022c). In particular, miRNA and mRNA sequencing showed that BEV from PM significantly affected apoptotic signaling pathways and mitochondrial function. Specifically, the expression of Bax, B-cell lymphoma-2, cytochrome C, and caspase-3 was upregulated, and miR-96-5p was downregulated, while intracellular ROS levels and mitochondrial membrane potential were increased. Moreover, OVX-induced bone loss in OP mice was reduced by PM-derived BEVs **(** Figure 3 **)**. The molecular mechanism of novel BEVs with GM osteoprotective activity was demonstrated by histological sequencing and applied to the clinical treatment of OP patients.

These findings revealed a novel way of gut-bone axis mediation by intestinal bacteria, providing the potential for the use of transgenes (particularly probiotics) and their functional BEVs for the treatment of OP and OP fractures. In addition, the usage of naturally occurring BEVs created by genetic engineering will grow in popularity as a field of study. Further enhancing the treatment efficacy of these BEVs for OP by the application of genetic engineering approaches.

### 6.1 BEVs future perspectives

The possibility of treating OP through natural BEVs was mentioned above, and the benefit of BEVs in the treatment of systemic skeletal disorders like OP has shown tremendous promise. BEV-based therapies are a more favorable strategy for OP than parental bacteria, and probiotics, in particular, have been shown to control the onset and disease progression. This is because of the nanosized structures, great biocompatibility, unique cell-free system, and non-reproducible properties of BEVs. By controlling the endocrine, immunological, and intestinal metabolites, the gut-bone axis has been identified as a possible target for the therapy of OP (Tyagi et al., 2012; Sharon et al., 2014; Zhang et al., 2021c). For the gut-bone axis, BEVs have steadily evolved into a very important communication tool. In light of this, probiotics such as *AKK, Bifidobacterium spp., and Lactobacillus spp*. have been demonstrated to work well against OP and release BEVs that may be more suitable for OP treatment.

Both organic and synthetic BEVs are possibilities for OP treatment. BEVs have developed gradually into a critical communication tool for the gut-bone axis. As a result, using naturally occurring BEVs made from these bacteria is a favored method for treating OP in addition to the documented microorganisms, particularly probiotics. Furthermore, two engineering techniques can be used to create therapeutic BEVs: parental engineering strains and engineering natural BEVs. The improved features of these modified BEVs, such as low toxicity, high yield, bone targeting capability, and bone healing capacity, enable a wider range of treatment options for OP.

These organic BEVs offer a great foundation for the management of OP. However, BEVs have several drawbacks, such as limited therapeutic efficiency and poor targeting capacity, which may necessitate substantial dosages in order to ensure efficacy. Thanks to developments in synthetic biology, it is now possible to adapt multifunctional strains to produce extremely effective BEVs for OP treatment. In addition to engineering the parental strains, BEV-based engineering approaches also provide a wide range of functionalities for BEVs. It is possible to create BEVs with a variety of capabilities (high productivity, low toxicity, bone-targeting ability, and bone-therapeutic ability), which is a viable platform for OP treatment, according to the engineering approaches for parental strains and BEVs. Currently, intravenous injection is the major method used to treat systemic disorders caused by BEVs (Chen et al., 2020c; Chen et al., 2022b; Li et al., 2022), and the only route of administration is rare (Liu et al., 2021b). Oral delivery is typically a safer therapeutic option than an intravenous injection, with higher patient compliance and reduced medical expenses (Taddio et al., 2012; Vela Ramirez et al., 2017). The complicated gastric environment limits the oral delivery of BEVs. Dopamine polymerization-mediated decoration was created by Liu et al. (Pan et al., 2021) to shield probiotics from bile salts and stomach acid. Similar decorating platforms can be created to guarantee BEVs’ oral effectiveness and increase their effectiveness in treating OP.

Interestingly, a team of researchers demonstrated an oral symbiotic bacterial approach based on BEVs (Yue et al., 2022). They found that by interacting with immune cells in the lamina propria, BEVs produced from intestinal bacteria can cross the intestinal epithelial barrier and result in immunomodulation. After oral treatment of intestinal commensal bacteria (with araBAD promoter) and arabinose, the bacteria in the intestine controlled the synthesis of BEVs with target antigens (araBAD promoter inducer). These modified BEVs distributed the stimulant chemicals after crossing the gut epithelial barrier. Similar research also showed that OP mice had significantly lower levels of immune cells called Treg, which have been shown to can improve bone mass and prevent OC development by downregulating the creation of RANKL (Zhu et al., 2020b; Ren et al., 2021), an important target in the development and activation of OCs (Wan et al., 2018; Chen et al., 2020d). Therefore, the administration of oral commensal bacteria approaches based on BEVs to give stimulatory compounds to increase Treg proliferation is an effective way for OP treatments (Campbell et al., 2020; Fu et al., 2022).

Notably, BEVs will be an effective tool for controlling OP fracture. Based on clinical findings, the research team proposed a “three-in-one” therapy strategy consisting of active anti-OP nanocarriers to optimize bone healing and improve bone implantation (Chen et al., 2022c). In addition to serving as anti-OP nanocarriers, BEVs can work in conjunction with various biomaterials to hasten the healing of OP fractured bones, including metal scaffolds, hydrogels, and mesoporous inorganic biomaterials (Mora-Raimundo et al., 2019; Mao et al., 2021; Zou et al., 2021). Despite ongoing difficulties, more studies into BEV-based treatments will likely result in cutting-edge treatments for OP and its side effects, speeding up their clinical translation use.

### 6.2 The use of exosomes in OA treatment

In the same way as the OP, Osteoarthritis (OA) is also a prevalent orthopaedic disease associated with aging. Recent studies have shown that they share similarities in pathological features and pathogenesis. Subchondral bone loss is a feature of OP and is also present in the early stages of OA, which suggests that OA can be attempted in the same way as OP to suppress the course of the disease (Bultink and Lems, 2013; Lafeber and van Laar, 2013). Therefore, as with OP, treatments for OA mediated by EVs show great potential. In the last few years, there has been increasing evidence that miRNA can also regulate genes in a non-cell-autonomous manner. Exosomes are *in vivo*-derived EVs that contain intact, mature 21 nt-miRNAs that act in intercellular communication. Exosome biogenesis is upregulated by various signals, and their secretion results from the passive activation of different mechanisms. Several parallel pathways can activate exosome biogenesis which releases from the passive activation of various signaling pathways. OCs secrete miR-214-rich exosomes for delivery to OBs in bone. Importantly, in human OA, miRNA is an important regulator of molecular pathophysiological processes in synovium and cartilage.

**FIGURE 4:**
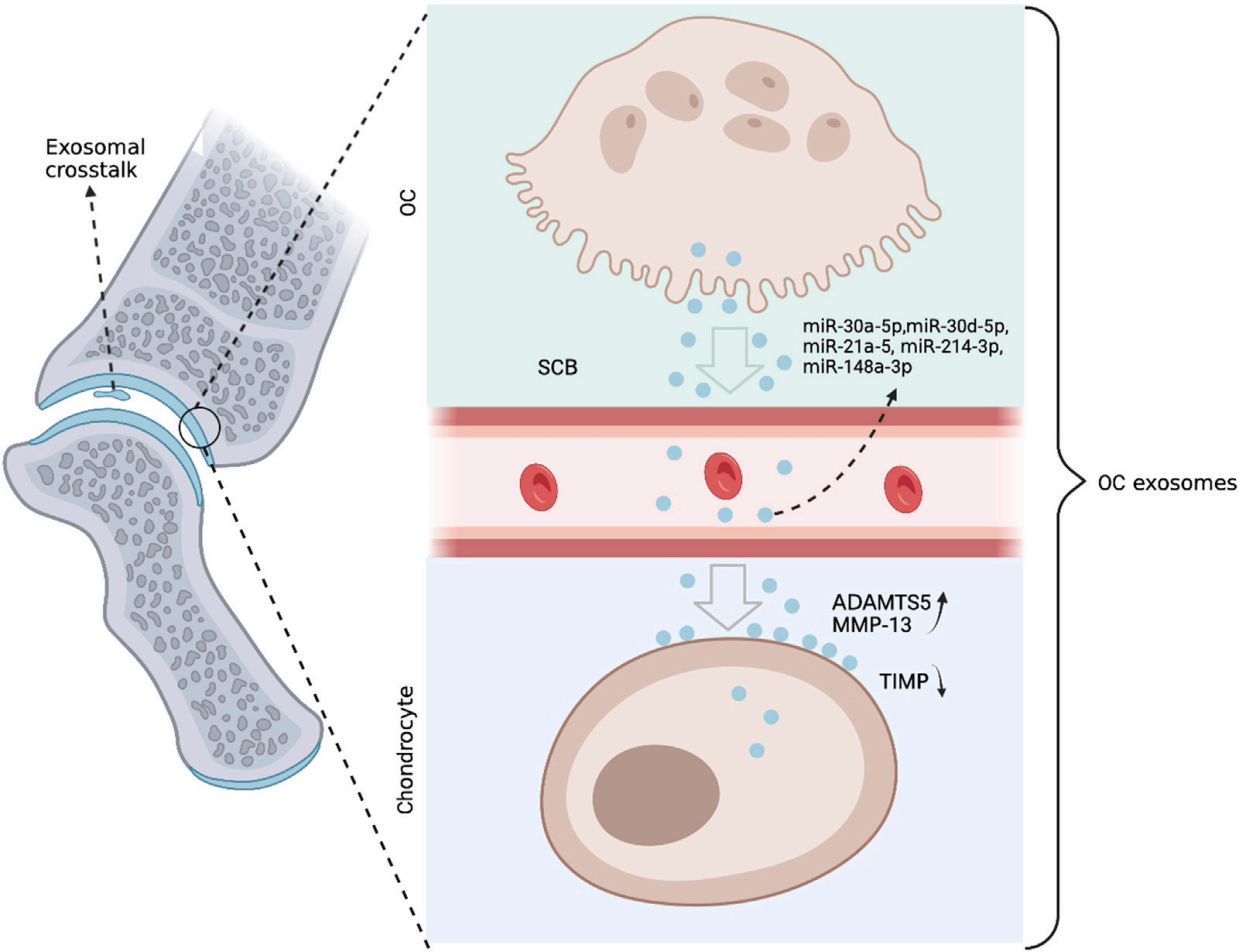
Anterior cruciate ligament transection (ACLT) was performed on mice to create the OA phenotype. Combined with *in vitro* culture models, small extracellular vesicles rich in coding miRNAs are secreted by OCs. These miRNAs are translocated to chondrocytes, where they suppress genes like TIMP that alleviate matrix deterioration, increasing the activities of ADAMTS5 and MMP-13. The concentration of OC miRNAs in circulating exosomes increases, and they appear to leave the SCB and re-enter the chondrocytes.

**FIGURE 5:**
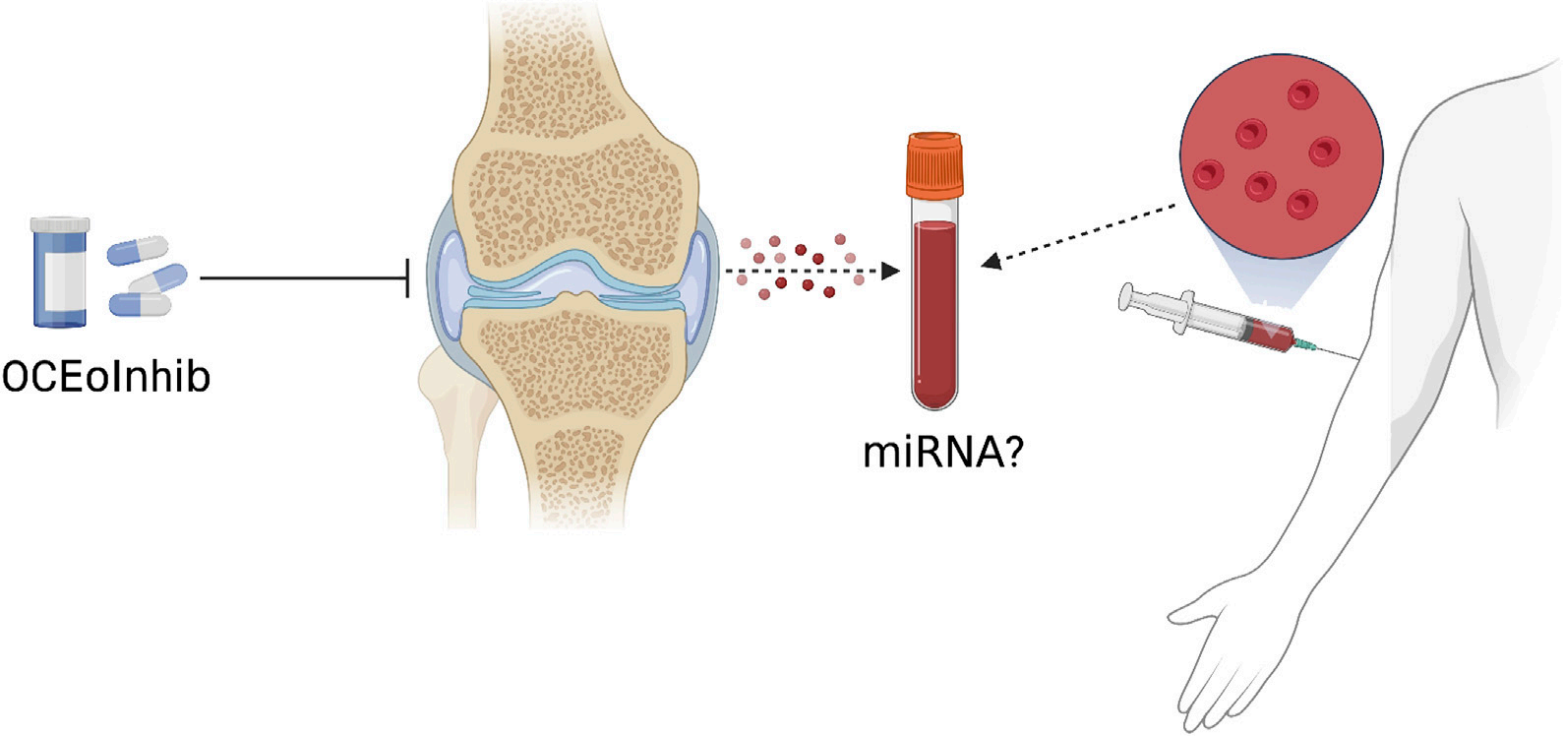
A new OC-targeted exosome inhibitor (OCEoInhib) to block Rab27-mediated OC exosome release. The measurement of OC miRNA in circulating exosomes could be used to diagnose illnesses and monitor their treatments.

A new study by Liu et al. shows that OC-derived miRNA is upregulated during surgically induced OA formation in mice *in vivo* (Liu et al., 2021c). They used an OC-targeted delivery system that disrupted miRNA biogenesis and exosome production in OC by eliminating Rab27a and the Dicer enzyme that controls miRNA production, significantly delaying OA progression **(** Figure 4 **)**. Nevertheless, questions remain about the mechanism of how OC exosomes affect chondrocytes. The authors showed that the expression level of miR-214 in OC was negatively correlated with the expression of metalloproteinase inhibitors in chondrocytes. Mechanistically, exosomal transfer of OC-miRNAs into chondrocytes reduces cartilage resistance to matrix degeneration, osteochondral angiogenesis, and sensory nerves during OA progression by inhibiting TIMP-2/3. The study by Liu et al. clearly shows that in injury-induced OA pathology, exosomes and miRNA play an important role in OC-chondrocyte crosstalk. Therefore, the transfer of exosomes of OC-miRNAs to chondrocytes is thought to be a viable treatment approach to alleviate the development of OA **(** Figure 5 **)**.

### 6.3 The future perspective of exosomes in OA treatment

The pathology of OA involves cartilage, subchondral bone, synovial tissues, and exosomes can act as hubs for their delivery pathways. Synovial fibroblast-derived exosomes can induce OA progression, and serum- and synovial-derived exosomes can contribute to the diagnosis of OA by increasing chondrocyte proliferation and suppressing inflammation. Remarkably Mφs-EXOs can repair joints or alleviate disease progression. While it is also true that these effects of exosomes will greatly facilitate research into the mechanisms, diagnosis, and treatment of OA, there are also several challenges. Firstly, exosomes contain many proteins and non-coding RNAs. Although they are involved in or regulate the development of OA, it is difficult to identify the exact molecules that contribute. Future studies could therefore focus on the functional dissection of a single component and the network of interactions between them. Secondly, both Mφs-EXOs and the modified exosomes act only in small animals, and the exact mechanisms are not yet understood. Therefore, their safety and efficacy *in vivo* need to be further investigated. In the future, they could be further tested on large animals.

## 7 Conclusion

Growing evidence points to mammalian and bacteria-derived EVs’ possibility of acting as nano messengers for communications between the host and the microbiota. BEVs represent their parental microbes in a range of interpersonal interactions. Contrary to their origin, they are less likely to enter circulation. They can transfer their contents to locations distant from the intestine, such as the bones. Instead of individual metabolites and secreted proteins, the contents of EVs are encased in bilayers as a means of protecting them from lysozyme and RNase in the extracellular environment and facilitating their diffusion to distant organs (Al-Nedawi et al., 2015; Choi et al., 2015). EVs are still underutilized as a means of connecting with the host.

Earlier investigations have examined the characterization of their proteome and/or RNA contents or the connection of EVs from a particular microorganism with certain bodily reactions (Perez-Burgos et al., 2013; Al-Nedawi et al., 2015; Emery et al., 2017; Zakharzhevskaya et al., 2017; Zhang et al., 2018; Lee et al., 2020). This might be explained by the absence of standardized techniques for isolating and identifying BEV components as well as by the absence of clear-cut biomarkers extracted from BEVs. Furthermore, host EVs and BEVs are not separated using the current techniques. Recent studies have revealed several methods for separating BEVs from bodily fluids using size exclusion chromatography, density gradient centrifugation, and ultrafiltration (Tulkens et al., 2020).

Moreover, the lack of accurate ways to determine the parental bacterial origin of various EVs is another challenge, BEVs or their observed content in different microbial communities, such as transgenic (Nahui Palomino et al., 2021). Future studies are necessary to demonstrate the relationship between the variability of the parent microbiota and the variability of BEV production and composition. Besides, more investigation is needed to determine how BEVs are packaged by microbial cells and the reasons behind the packaging of these specific molecules. Questions such as whether they target specific cells or host cells, whether they can cross biological barriers, including the blood-brain barrier and the intestinal barrier, how they target host cells, and how they release their contents remain to be investigated.

Furthermore, the study on targeting exosome-mediated pathogenesis of other bone disorders like OA is unique in its molecular penetration, clearly demonstrating that exosomes and miRNAs have an important role in OC-chondrocyte crosstalk in injury-induced OA pathology (Meulenbelt et al., 2021). It will be important to investigate in the future whether there is an association between exosome crosstalk on the development of age-related OA. From experiments in mice, it is shown that tissue-targeted inhibition of disease expression by exosomes is feasible. It could lead to breakthroughs in the treatment of OA and other orthopedic diseases. Although the observation of elevated serum exosome levels in patients with severe knee injuries increases the translational significance of this finding, the pathological process of OA characterized by ACLT in young mice is not similar to that of age-related OA in humans.

The recent developments discussed in this review give us a glimpse of host and bacteria EVs’ evolving role as mediators of host-microbiota interaction, despite the various challenges that must be overcome before they may possibly be exploited as a platform for the delivery of biologic therapeutics to particular body sites.

## Glossary

OP: Osteoporosis
EVs: Extracellular vesicles
BEVs: Bacterial extracellular vesicles
OA: Osteoarthritis
GM: Gut microbiota
OBs: osteoblasts
OCs: osteoclasts
MSCs: Mesenchymal stem cells
HucMSC: Human umbilical cord MSC
hiPSC-MSC: human induced pluripotent stem cells
BMP: Bone morphogenetic protein
PI3K: Phosphoinositide-3 kinase
BMSCs: Bone marrow mesenchymal stem cells
RANKL: Receptor activator of nuclear factor-κB ligand
OPG: Osteoclastogenesis inhibitory factor
RANK: Receptor activator of nuclear factor-κB
Runx2: Runt-related transcription factor 2
YAP1: Yes-associated protein 1
MT1DP: Metallothionein 1D pseudogene
PTEN: phosphatase and tensin homolog
Mφs: Macrophages
DUSP1: Ddual specificity phosphatase 1
JNK: C-Jun N-terminal kinase
EC: Endothelial cells
EPCs: Endothelial progenitor cells
MALAT-1: Metastasis associated lung adenocarcinoma transcript 1
SCFAs: Short-chain fatty acids
FAO: Food and Agriculture Organization of the United Nations
WHO: World Health Organization
MEVs: Mammalian EVs
TLR: Toll-Like Receptors
LGG: *Lactobacillus* rhamnosus GG
AKK: Akkermansia muciniphila
FMT: Fecal microbiota transplantation
EGM: Elderly GM
CGM: Children GM
OVX: Ovariectomy
PM: *Proteus mirabilis*
Bax: BCL2-associated X protein
ROS: Reactive oxygen species
TIMP: tissue inhibitor of metalloproteinases
SCB: subchondral bone
ACLT: anterior cruciate ligament transection
ADAMST5: activity of a disintegrin and metalloproteinase with thrombospondin motifs 5
MMp-13: matrix metalloproteinase-13
